# Cu/TiO_2_ Derived from Cu-Doped MIL-125 for Enhanced Photocatalytic CO_2_-to-CH_4_ Conversion

**DOI:** 10.3390/molecules31132304

**Published:** 2026-07-01

**Authors:** Haopeng Cui, Zhiying Li, Siyu Huang, Tianyi Zhang, Xiaodong Zhang, Zhongxiao Zhang, Jianqiu Lei, Ning Liu

**Affiliations:** 1 School of Environment and Architecture, University of Shanghai for Science and Technology, Shanghai 200093, China; 2 Shanghai Noncarbon Energy Conversion and Utilization Institute, Shanghai 200240, China; 3 College of Smart Energy, Shanghai Jiao Tong University, Shanghai 200240, China; 4 Shanghai Institute of Optics and Fine Mechanics, Chinese Academy of Sciences, Shanghai 201800, China

**Keywords:** Cu/TiO_2_, photocatalytic CO_2_ reduction, CH_4_ selectivity, MOF-derived photocatalyst

## Abstract

Photocatalytic CO_2_ reduction into CH_4_ is a promising route for solar fuel production, but its efficiency is still limited by poor charge separation, insufficient CO_2_ activation, and sluggish multi-electron transfer kinetics. Herein, Cu-modified TiO_2_ (Cu/TiO_2_) was prepared by calcining a Cu-modified defective MIL-125(Ti) precursor, denoted as Cu-MIL-125, through a temperature-controlled calcination strategy. The effects of calcination temperature on the structural evolution, surface chemical states, interfacial charge transport, and CO_2_ photoreduction performance were examined. These results indicated that the Cu/TiO_2_ was successfully prepared, while the crystallinity, porous structure, and interfacial electronic properties of Cu/TiO_2_ were strongly dependent on the calcination temperature. Among the obtained samples, the Cu/TiO_2_ sample obtained by calcining Cu-MIL-125 at 450 °C (450 Cu/TiO_2_) exhibited the highest CH_4_ formation rate, reaching 15.90 μmol g^−1^ h^−1^, corresponding to an approximately 9.8-fold enhancement over TiO_2_ calcined from defective MIL-125(Ti) at 450 °C, together with a high CH_4_ selectivity of 93.05%. Control experiments and ^13^CO_2_ isotope-labeling tests confirmed that the detected carbon-containing products were generated from CO_2_ under photocatalytic conditions. In situ diffuse reflectance infrared Fourier transform spectroscopy measurements further revealed the formation of carbonate, bicarbonate and hydrogenated carbon-containing intermediates during the reaction. This work offers a practical route for constructing metal–organic framework-derived Cu/TiO_2_ photocatalysts for selective CH_4_ production from CO_2_.

## 1. Introduction

The continuous increase in atmospheric carbon dioxide (CO_2_) concentration, mainly originating from the extensive consumption of fossil fuels, has caused growing concerns regarding climate change and sustainable carbon utilization [1]. Solar-driven CO_2_ photoreduction offers a mild route for transforming CO_2_ into useful fuels and chemicals [2]. Among the possible products, carbon monoxide (CO) and methane (CH_4_) are particularly attractive because they connect CO_2_ utilization with renewable energy storage [3]. However, efficient CO_2_ photoreduction remains difficult because CO_2_ is highly stable, multi-electron transfer is kinetically slow, and photogenerated carriers tend to recombine rapidly [4]. In particular, the selective formation of CH_4_ is more challenging than CO generation because it involves an eight-electron reduction pathway and requires the stabilization of successive reaction intermediates [5,6]. Therefore, photocatalysts for CH_4_-selective CO_2_ reduction should combine effective charge separation, accessible active sites, and suitable surface reaction behavior. Meanwhile, reliable evaluation of heterogeneous photocatalytic CO_2_ reduction also requires clear reporting of experimental conditions, activity metrics, catalyst stability, data reproducibility, and mechanistic evidence [7].

Titanium dioxide (TiO_2_) is a representative semiconductor photocatalyst because of its chemical stability, low cost, low toxicity, and appropriate band-edge positions for CO_2_ reduction [8,9,10]. Nevertheless, pristine TiO_2_ is restricted by its wide band gap, weak visible-light utilization, limited CO_2_ adsorption/activation, and fast carrier recombination, which together lead to low activity and poor product selectivity [11,12]. Therefore, rational modification of TiO_2_ is required to simultaneously improve light utilization, charge separation, surface active-site exposure, and interfacial CO_2_ activation [13]. The photocatalytic behavior of TiO_2_ is strongly influenced by its crystal phase, crystallinity, defect structure, surface hydroxylation, and porosity, all of which are closely related to the preparation route and thermal treatment [14]. Among various modification strategies, constructing TiO_2_ from metal–organic framework (MOF) precursors offers an effective route to regulate the morphology and porosity of TiO_2_, thereby exposing more active sites and facilitating charge separation [15,16,17]. In addition, MOFs with designable pore structures can provide confined environments for accommodating metal species and promoting the adsorption and activation of reactant molecules [18]. Among Ti-based MOFs, Materials of Institut Lavoisier-125(Ti) (MIL-125(Ti)) is particularly attractive due to its Ti-oxo clusters and organic linkers, which provide a well-defined framework for subsequent structural transformation. After thermal conversion, MIL-125(Ti)-derived TiO_2_ can inherit part of the porous architecture and morphology of the MOF precursor, thereby increasing accessible surface area, exposing more surface sites, and facilitating CO_2_ adsorption and diffusion [15,19]. Nevertheless, the calcination temperature critically affects the MOF-to-TiO_2_ conversion, the crystallinity of TiO_2_, and the retention of porous structures [20,21]. Insufficient calcination may lead to incomplete framework decomposition and poor TiO_2_ crystallization, whereas excessive calcination can cause pore collapse, particle sintering, and loss of accessible active sites [22]. Although MOF-derived TiO_2_ can provide improved porosity and surface properties, electronic tuning is still needed to reduce charge recombination and promote selective CO_2_-to-CH_4_ conversion [9,23].

Introducing Cu species is a useful approach for adjusting the electronic structure and surface reaction behavior of TiO_2_-based photocatalysts [23]. The introduction of Cu species can facilitate interfacial electron transfer and suppress charge recombination by serving as electron-trapping or charge-transfer sites. Aguirre et al. reported Cu_2_O/TiO_2_ heterostructures for photocatalytic CO_2_ reduction, in which interfacial charge transfer promoted CO formation and TiO_2_ protected Cu_2_O from photocorrosion [24]. These studies indicate that Cu–TiO_2_ interfacial regulation is an effective strategy for CO_2_ photoreduction, whereas further exploration of precursor-derived Cu distribution and Cu–TiO_2_ interfacial interaction remains important for promoting CH_4_-selective CO_2_ reduction. In addition, Cu sites may strengthen CO_2_ adsorption/activation and adjust the interaction with reaction intermediates, which is beneficial for CH_4_ generation during photocatalytic CO_2_ reduction [25,26]. After calcination, Cu-modified MIL-125(Ti) can be converted into Cu-modified TiO_2_ (Cu/TiO_2_), while the chemical state, dispersion, and interfacial interaction of Cu species are highly dependent on the calcination temperature [15,22].

In this study, defective MIL-125(Ti) (MIL-125) was first synthesized, followed by the introduction of Cu to prepare Cu-modified defective MIL-125(Ti) (Cu-MIL-125). Cu/TiO_2_ was then prepared using Cu-MIL-125 as the precursor, with the properties of Cu/TiO_2_ finely tuned via the calcination temperature optimization. Different from conventional Cu/TiO_2_ modification strategies based on post-impregnation, deposition, or direct coupling with preformed copper oxides, this precursor-derived route allows Cu species to be introduced before thermal conversion, which is beneficial for regulating Cu dispersion and Cu/TiO_2_ interfacial interaction. The relationships between thermal transformation, interfacial electronic structure, and photocatalytic CO_2_ reduction performance were systematically investigated. Furthermore, in situ diffuse reflectance infrared Fourier transform spectroscopy (DRIFTS) and isotope-labeling experiments were performed to elucidate the reaction pathway and verify the carbon source of CH_4_. This study presents a strategy for constructing MOF-derived TiO_2_ photocatalysts for CO_2_ photoreduction, and demonstrates that the photocatalytic performance of Cu/TiO_2_ can be effectively optimized through the control of calcination conditions.

## 2. Results and Discussion

### 2.1. Structural Characterization of Cu/TiO_2_ Photocatalysts

**Figure 1:**
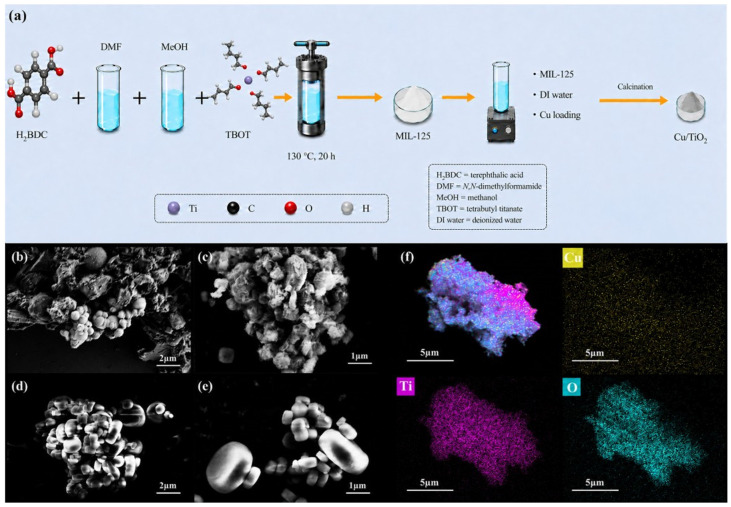
(**a**) Schematic illustration of the synthesis process of Cu/TiO_2_. SEM images of (**b**) MIL-125, (**c**) Cu-MIL-125, and (**d**,**e**) 450 Cu/TiO_2_. (**f**) EDS elemental mapping images of 450 Cu/TiO_2_.

The synthesis process of Cu/TiO_2_ is illustrated in Figure 1a. The morphologies of MIL-125, Cu-MIL-125, and the derived 450 Cu/TiO_2_ samples were investigated by scanning electron microscopy (SEM). As shown in Figure 1b,c, after the introduction of Cu, Cu-MIL-125 retained a similar overall morphology to MIL-125, suggesting that Cu did not lead to obvious collapse of the precursor structure [23]. After calcination, the obtained 450 Cu/TiO_2_ consisted of irregular particles and displayed a disk-like aggregated morphology (Figure 1d,e), indicating the transformation of the MOF precursor into TiO_2_-based particles [27]. Energy-dispersive X-ray spectroscopy (EDS) mapping (Figure 1f) showed that Ti, O, and Cu were all detected in the 450 Cu/TiO_2_ sample, and their signals were observed in the selected region, which confirmed the successful introduction of Cu into the 450 Cu/TiO_2_. No obvious large Cu-rich aggregates were observed in the mapping images, indicating that Cu species were distributed without apparent microscale aggregation within the spatial resolution of SEM-EDS [28].

**Figure 2:**
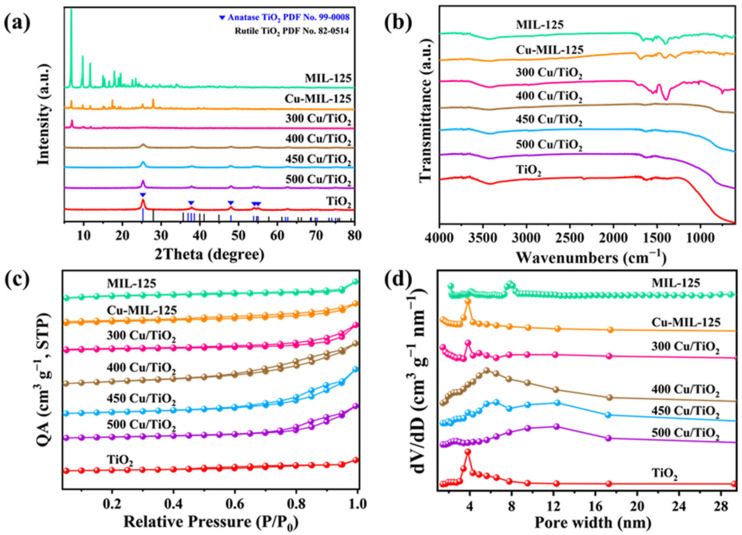
(**a**) XRD patterns, (**b**) FT-IR spectra, (**c**) N_2_ adsorption–desorption isotherms, and (**d**) pore-size distribution curves of the prepared samples.

Figure 2a presents the X-ray diffraction (XRD) patterns of MIL-125, Cu-MIL-125, TiO_2_ (derived from MIL-125 at 450 °C) and Cu/TiO_2_ samples. MIL-125 and Cu-MIL-125 showed the typical diffraction features of the MIL-125(Ti) framework, suggesting the successful preparation of the MOF precursors [29,30]. After calcination, the characteristic diffraction peaks of the MIL-125(Ti) framework gradually disappeared, accompanied by the formation of TiO_2_-related diffraction features. The diffraction peaks of TiO_2_ and the calcined Cu/TiO_2_ samples located at 2θ = 25.3°, 37.8°, 48.0°, 53.9°, and 55.1° could be assigned to the (101), (004), (200), (105), and (211) planes of anatase TiO_2_, respectively, indicating the formation of anatase TiO_2_ after calcination [31]. At 300 °C, TiO_2_-related diffraction peaks of anatase TiO_2_ were still weak, indicating that the thermal conversion of the Cu-MIL-125-derived precursor was incomplete and that well-crystallized TiO_2_ had not yet formed. With increasing calcination temperature, the characteristic peaks of anatase TiO_2_ became more pronounced, suggesting the gradual crystallization of TiO_2_ during the MOF-derived thermal transformation. No obvious diffraction peaks assigned to crystalline Cu or CuO_x_ species were detected, suggesting that Cu species were dispersed or present in a low-crystallinity form below the detection limit of XRD.

Fourier transform infrared spectroscopy (FT-IR) spectra were used to follow the changes in chemical bonds and functional groups during the thermal conversion process, as shown in Figure 2b. For MIL-125 and Cu-MIL-125, the bands near 1600 and 1400 cm^−1^ were associated with the stretching modes of carboxylate groups in terephthalate ligands, verifying the retained MIL-125(Ti) framework [32,33]. After calcination, these ligand-related bands gradually weakened with increasing temperature, suggesting the progressive decomposition and removal of organic ligands. Meanwhile, a broad band around 650 cm^−1^, corresponding to Ti-O-Ti stretching vibration [34], became more pronounced in the calcined samples, indicating the formation of TiO_2_-based structures. Combined with the results of XRD, the weakened organic-ligand signals and the enhancement of Ti-O-Ti vibration suggested the gradual conversion of Cu-MIL-125 into TiO_2_ with improved crystallinity at higher calcination temperatures.

The textural evolution of MIL-125, Cu-MIL-125, TiO_2_ and Cu/TiO_2_ samples was analyzed from N_2_ adsorption–desorption isotherms (Figure 2c,d). All the samples displayed type IV adsorption–desorption isotherms, suggesting mesoporous structures of these samples [27]. Correspondingly, the pore-size distribution curves in Figure 2d further confirmed the mesoporous features of these samples. MIL-125 exhibited a high Brunauer–Emmett–Teller (BET) surface area of 763.48 m^2^ g^−1^. After Cu incorporation, the value declined to 135.05 m^2^ g^−1^ (Table 1). For comparison, the TiO_2_ sample derived from MIL-125 at 450 °C without Cu introduction showed a BET surface area of 44.28 m^2^ g^−1^, a total pore volume of 0.11 cm^3^ g^−1^, and an average pore size of 10.07 nm. For the calcined Cu/TiO_2_ samples, a clear temperature-dependent textural evolution was observed: the BET surface area declined from 164.35 to 66.64 m^2^ g^−1^ as the calcination temperature increased from 300 to 500 °C, whereas the average pore size enlarged from 7.27 to 18.89 nm. This result suggested that higher calcination temperatures can increase the pore size of Cu/TiO_2_ [35,36]. These textural parameters suggest that Cu introduction and calcination temperature affected the porous structure of the MOF-derived TiO_2_-based samples, while the photocatalytic performance should be considered together with crystallinity, interfacial Cu regulation, and charge-transfer behavior.

**Figure 3:**
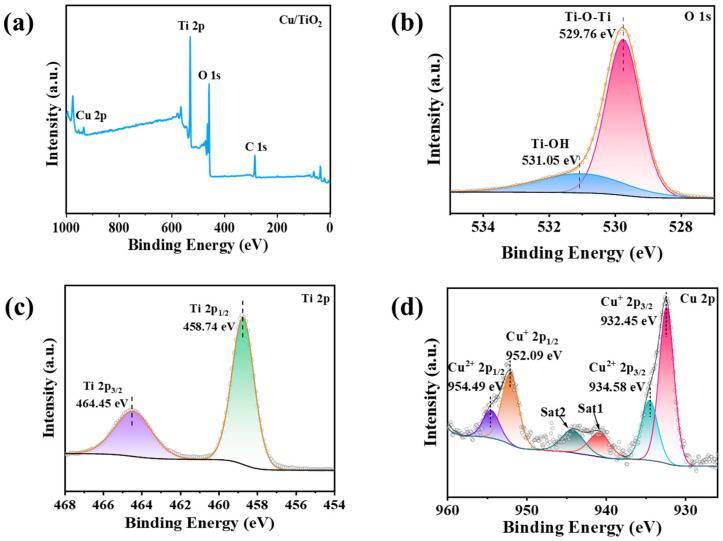
XPS spectra of 450 Cu/TiO_2_: (**a**) survey, (**b**) O 1s, (**c**) Ti 2p, and (**d**) Cu 2p.

The surface chemical environment of 450 Cu/TiO_2_ was further probed by X-ray photoelectron spectroscopy (XPS), as shown in Figure 3. The survey spectrum (Figure 3a) showed the signals of C, O, Ti, and Cu, consistent with the elemental distribution observed from EDS mapping. In the Ti 2p spectrum (Figure 3c), two peaks located at 458.92 and 464.65 eV corresponded to Ti 2p_3/2_ and Ti 2p_1/2_ of Ti^4+^, respectively [37]. The O 1s spectrum was fitted with two components at 529.76 and 531.05 eV (Figure 3b), corresponding to lattice oxygen in the Ti-O-Ti and surface Ti-OH species, respectively [31]. For Cu 2p (Figure 3d), the peaks at 932.45 and 952.09 eV were attributed to Cu^+^ species [23], while those at 934.58 and 954.49 eV were assigned to Cu^2+^ species [31]. The satellite features further supported the presence of Cu^2+^ [37,38,39]. The Cu 2p XPS results indicate the coexistence of Cu^+^ and Cu^2+^ species on the surface of 450 Cu/TiO_2_, suggesting a mixed-valence Cu surface environment.

### 2.2. Photocatalytic CO_2_ Reduction Performance

**Figure 4:**
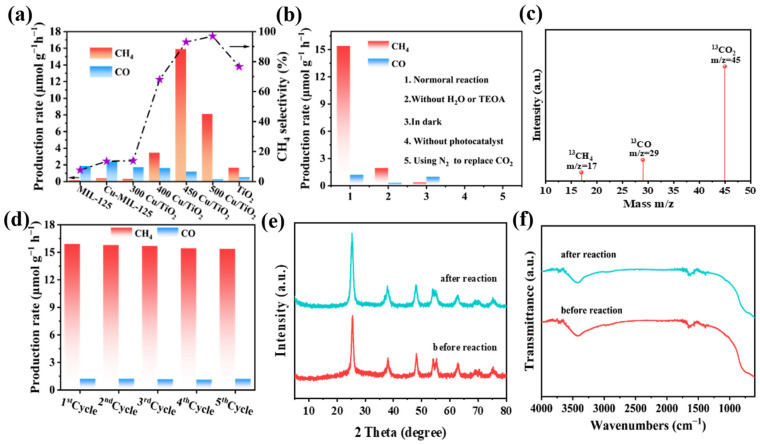
Photocatalytic CO_2_ reduction performance and stability evaluation. (**a**) Production rates of products and CH_4_ selectivity over different samples. (**b**) Control experiments under different reaction conditions. (**c**) Mass spectral signals of ^13^CH_4_ and ^13^CO obtained from the ^13^CO_2_ isotope-labeling test. (**d**) Cycling stability of 450 Cu/TiO_2_ for photocatalytic CO_2_ reduction. (**e**) XRD patterns and (**f**) FT-IR spectra of 450 Cu/TiO_2_ before and after the photocatalytic reaction.

The CO_2_ photoreduction activity of different samples was compared by monitoring the formation of CH_4_ and CO. As shown in Figure 4a, pristine TiO_2_, MIL-125, and Cu-MIL-125 showed relatively low formation rates of CH_4_, indicating the limited ability to promote CH_4_ generation from CO_2_. By contrast, the Cu/TiO_2_ samples exhibited clearly enhanced photocatalytic activity, demonstrating that the calcined Cu/TiO_2_ exhibited excellent performance for the photocatalytic reduction of CO_2_ to CH_4_. For Cu/TiO_2_, the CH_4_ production rate increased markedly as the calcination temperature increased from 300 to 450 °C. Among them, 450 Cu/TiO_2_ exhibited the highest CH_4_ production rate of 15.90 μmol g^−1^ h^−1^, which was 106.0, 43.0, and 9.8 times those of MIL-125, Cu-MIL-125, and pristine TiO_2_, respectively. Meanwhile, 450 Cu/TiO_2_ maintained a high CH_4_ selectivity of 93.05%, suggesting that the optimized calcination temperature enhanced the overall CO_2_ reduction activity and promoted the selective formation of CH_4_. The superior performance of 450 Cu/TiO_2_ can be related to the appropriate structural evolution during calcination. Calcination at 450 °C may improve the crystallinity of anatase TiO_2_ and strengthen the interaction between Cu species and TiO_2_. These changes could improve the utilization of photogenerated charges and provide more accessible reaction sites for CO_2_ activation and subsequent multi-electron conversion toward CH_4_. When the calcination temperature was further increased to 500 °C, the CH_4_ selectivity increased slightly to 96.93%, whereas the CH_4_ formation rate decreased to 8.09 μmol g^−1^ h^−1^. This result may be related to the reduced specific surface area and possible changes in the porous structure and interfacial electronic properties at higher calcination temperature, indicating that the photocatalytic performance was governed by the combined effects of crystallinity, porosity, and Cu/TiO_2_ interfacial regulation rather than the specific surface area alone. Overall, the 450 Cu/TiO_2_ sample achieved a CH_4_ formation rate of 15.90 μmol g^−1^ h^−1^ with a CH_4_ selectivity of 93.05%, suggesting that the Cu-MIL-125-derived strategy is effective for promoting selective CO_2_-to-CH_4_ conversion. A comparison with representative TiO_2_-based photocatalysts reported for CO_2_-to-CH_4_ reduction is summarized in Table 2, further highlighting the relatively high CH_4_ formation rate of 450 Cu/TiO_2_.

Control experiments were performed to verify the essential factors during the photocatalytic CO_2_ reduction reaction (Figure 4b). In contrast, the formation of CH_4_ and CO was markedly suppressed in the absence of H_2_O, TEOA, light irradiation, photocatalyst, or CO_2_. These results confirmed that light irradiation, CO_2_, H_2_O and TEOA are indispensable for the photocatalytic CO_2_ reduction process. A ^13^CO_2_ isotope-labeling experiment was conducted to identify the carbon source of the products (Figure 4c). The signals assigned to ^13^CH_4_ and ^13^CO were observed at *m*/*z* = 17 and 29, respectively, confirming that the detected carbon-containing products originated from CO_2_ photoreduction rather than from residual carbon species or catalyst decomposition.

The stability of 450 Cu/TiO_2_ was evaluated by five consecutive cycling tests (Figure 4d). The CH_4_ and CO formation rates showed no obvious decrease after repeated cycles, suggesting a good photocatalytic stability of 450 Cu/TiO_2_. Moreover, the XRD patterns (Figure 4e) and FT-IR spectra (Figure 4f) of 450 Cu/TiO_2_ before and after the reaction showed no significant changes, further confirming its structural stability during the photocatalytic process.

### 2.3. Mechanism of Photocatalytic Activity Enhancement

To gain insight into the factors responsible for the improved CO_2_ photoreduction activity, ultraviolet–visible diffuse reflectance spectroscopy (UV-vis DRS), Tauc plots, Mott–Schottky analysis, photoluminescence (PL) spectroscopy, transient photocurrent measurements, and electrochemical impedance spectroscopy (EIS) were employed to investigate the optical response, electronic band structure, and charge-carrier separation and transfer behaviors of the samples.

**Figure 5:**
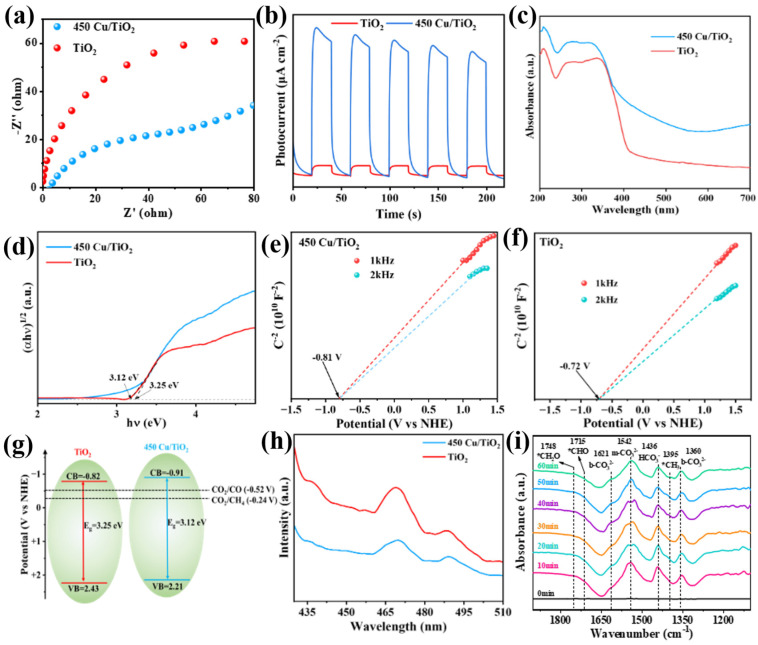
Charge-transfer behavior, optical properties, band structures, and surface-intermediate evolution of TiO_2_ and 450 Cu/TiO_2_: (**a**) EIS Nyquist plots, (**b**) transient photocurrent responses, (**c**) UV-vis diffuse reflectance spectra, (**d**) Tauc plots, Mott–Schottky plots of (**e**) TiO_2_ and (**f**) 450 Cu/TiO_2_, (**g**) estimated band structures of TiO_2_ and 450 Cu/TiO_2_ based on Mott–Schottky and Tauc analyses, (**h**) PL spectra, and (**i**) in situ DRIFTS spectra of CO_2_ photoreduction over 450 Cu/TiO_2_.

As shown in Figure 5a, 450 Cu/TiO_2_ exhibited much smaller arc radii than pristine TiO_2_, indicating improved interfacial charge transport after Cu modification, which was favorable for photocatalytic CO_2_ reduction [31].

Transient photocurrent measurements were conducted to compare the photoinduced charge-transport behavior of TiO_2_ and 450 Cu/TiO_2_. As shown in Figure 5b, 450 Cu/TiO_2_ exhibited higher photocurrent response than TiO_2_ under intermittent light irradiation. The stable and reproducible photocurrent response during repeated cycles further suggested the favorable photoelectrochemical stability of 450 Cu/TiO_2_. These results confirmed the improved charge separation and migration efficiency of 450 Cu/TiO_2_, which was beneficial for photocatalytic CO_2_ reduction.

UV-vis DRS, Tauc plots, and Mott–Schottky measurements were used to analyze the optical response and band positions of TiO_2_ and 450 Cu/TiO_2_. As shown in Figure 5c, pristine TiO_2_ showed an absorption edge around 400 nm in the near-UV region, corresponding to the wide-band-gap feature of anatase TiO_2_ [44]. Compared with TiO_2_, 450 Cu/TiO_2_ showed enhanced absorption in the visible-light region, indicating that the introduction of Cu species broadens the spectral response and improves the light-harvesting capability of TiO_2_ [23]. According to the corresponding Tauc plots (Figure 5d), the apparent optical band gaps of both TiO_2_ and 450 Cu/TiO_2_ were estimated to be 3.25 and 3.12 eV, respectively. The lower apparent optical band gap estimated for 450 Cu/TiO_2_ is consistent with its enhanced visible-light absorption after Cu introduction. Overall, these results indicated that Cu introduction modified the optical absorption behavior of TiO_2_, especially in the visible-light region.

Mott–Schottky measurements at 1 and 2 kHz were used to estimate the band-edge positions of TiO_2_ and 450 Cu/TiO_2_. As shown in Figure 5e,f, both TiO_2_ and 450 Cu/TiO_2_ displayed upward linear regions in the Mott–Schottky plots, indicating n-type behavior [45]. The flat-band potentials derived from the intercepts were −0.72 and −0.81 V versus the normal hydrogen electrode (NHE) for TiO_2_ and 450 Cu/TiO_2_, respectively. For n-type semiconductors, the conduction band edge is commonly approximated as 0.1 V more negative than the flat-band potential [46]. Accordingly, the conduction band positions were estimated as −0.82 V for TiO_2_ and −0.91 V for 450 Cu/TiO_2_ vs NHE. Using the Tauc-derived band gaps of 3.25 and 3.12 eV, the valence-band potentials (E_VB_) were obtained as 2.43 and 2.21 V vs NHE for TiO_2_ and 450 Cu/TiO_2_, respectively, based on E_VB_ = E_CB_ + E_g_, where E_CB_ and E_g_ represent the conduction-band potential and band gap, respectively. The more negative conduction band position of 450 Cu/TiO_2_ indicated a stronger reduction ability of photogenerated electrons, which was favorable for photocatalytic CO_2_ reduction [47]. Therefore, Cu modification may alter the estimated band structure of TiO_2_ and improve visible-light utilization, which could contribute to CO_2_ photoreduction together with the enhanced charge-separation behavior. PL spectra were recorded to compare the recombination behavior of photogenerated carriers (Figure 5h). The visible PL emission is mainly associated with radiative recombination through defect or surface-related states rather than direct band-to-band emission. The 450 Cu/TiO_2_ exhibited a much lower PL emission intensity than pristine TiO_2_, indicating reduced photogenerated carrier recombination. This result suggests that Cu modification and optimized calcination promoted charge separation in TiO_2_, which was consistent with previous reports that electronic-structure regulation can facilitate charge-transfer dynamics in MOF-based photocatalytic CO_2_ reduction systems [48].

In situ DRIFTS measurements were conducted to further investigate the reaction intermediates in the process of photocatalytic CO_2_ reduction over 450 Cu/TiO_2_ (Figure 5i). After CO_2_ and H_2_O adsorption in the dark, the sample was irradiated and the spectral changes were recorded as a function of irradiation time. The signals at 1542 cm^−1^ and 1621 cm^−1^ were attributed to monodentate carbonate (m-CO_3_^2−^) and bidentate carbonate (b-CO_3_^2−^), respectively, suggesting that CO_2_ was adsorbed and activated on the catalyst surface [49]. The peak at 1436 cm^−1^ corresponded to HCO_3_^−^, and the intensity gradually decreased with irradiation time, while the carbonate-related bands remained relatively unchanged [50]. This suggested that HCO_3_^−^ is a more reactive surface intermediate and may participate more readily in the subsequent reduction steps. In addition, the signals located at 1715 and 1748 cm^−1^ were associated with *CHO and *CH_2_O intermediates, respectively. Both of them are crucial hydrogenated intermediates for CH_4_ formation [50]. The peak at 1395 cm^−1^ related to the *CH_3_ further supported the progressive hydrogenation pathway from CO_2_-derived intermediates toward CH_4_ [51]. These DRIFTS results suggest a possible CO_2_-to-CH_4_ pathway involving initial CO_2_ adsorption and activation as carbonate/bicarbonate species, followed by stepwise hydrogenation through *CHO, *CH_2_O, and *CH_3_ intermediates toward CH_4_. This DRIFTS-supported pathway indicates that 450 Cu/TiO_2_ may promote CO_2_ activation and the formation of key hydrogenated intermediates, thereby contributing to its enhanced CH_4_ selectivity.

Combined with the EIS, photocurrent, UV-vis DRS, Mott–Schottky, and PL results, these observations indicated that the enhanced CO_2_-to-CH_4_ performance of 450 Cu/TiO_2_ is closely related to its calcination-regulated porous anatase structure, Cu-related interfacial electronic regulation, improved charge separation/transfer, and promoted CO_2_ activation.

## 3. Experimental Sections

### 3.1. Chemicals

Terephthalic acid (H_2_BDC, C_8_H_6_O_4_, ≥99.0%), N,N-dimethylformamide (DMF, C_3_H_7_NO, analytical reagent), absolute ethanol (C_2_H_6_O, analytical reagent), triethanolamine (TEOA, C_6_H_15_NO_3_, analytical reagent), methanol (CH_3_OH, analytical reagent), and copper(II) chloride dihydrate (CuCl_2_·2H_2_O, ≥99.0%) were supplied by Sinopharm Chemical Reagent Co., Ltd. (Shanghai, China). Tetrabutyl titanate (TBOT, C_16_H_36_O_4_Ti, analytical reagent) was obtained from Aladdin Chemical Reagent Co., Ltd. (Shanghai, China). All chemicals were directly used without additional purification.

### 3.2. Preparation of Photocatalyst

#### 3.2.1. MIL-125

MIL-125 was prepared through a solvothermal route [30]. Briefly, H_2_BDC (3.00 g) was first dissolved in a mixed solvent containing 54.0 mL of DMF and 6.0 mL of methanol under stirring. TBOT (1.2 mL) was then introduced as the titanium source. After stirring for several minutes, the solution was then sealed in a Teflon-lined stainless-steel autoclave and heated at 130 °C for 20 h. The resulting precipitate was collected by centrifugation, washed alternately with DMF and methanol, and finally dried under vacuum at 80 °C for 12 h to obtain MIL-125.

#### 3.2.2. Cu-MIL-125

Cu-MIL-125 was obtained by introducing Cu species into MIL-125 using a wet impregnation process [30]. In brief, 0.50 g of MIL-125 was dispersed in 40.0 mL of deionized water, followed by the addition of 9.96 mg of CuCl_2_·2H_2_O. The suspension was magnetically stirred for 3 h to allow sufficient contact between the precursor and Cu species. The product was then separated by centrifugation, rinsed with deionized water, and dried at 80 °C for 12 h.

#### 3.2.3. Cu/TiO_2_

Cu/TiO_2_ was obtained by calcining Cu-MIL-125 as a precursor in air at different temperatures (300, 400, 450, and 500 °C) for 4 h. The resulting samples were denoted as 300 Cu/TiO_2_, 400 Cu/TiO_2_, 450 Cu/TiO_2_, and 500 Cu/TiO_2_, respectively.

### 3.3. Characterization

The structure, composition, optical response, and charge-transfer properties of the samples were examined using a series of characterization techniques. The crystalline phases were identified from XRD (D8 ADVANCE, Bruker AXS GmbH, Karlsruhe, Germany) patterns recorded with Cu Kα radiation. FT-IR spectra were collected using a Nicolet iS50 FT-IR spectrometer (Thermo Fisher Scientific, Waltham, MA, USA) to identify surface functional groups and bonding characteristics. Sample morphology and elemental distribution were observed by SEM equipped with EDS (GEMINI 300, Carl Zeiss Microscopy GmbH, Oberkochen, Germany). N_2_ adsorption–desorption isotherms were measured using an Autosorb iQ2 analyzer (Quantachrome Instruments, Boynton Beach, FL, USA), and the BET specific surface areas, total pore volumes, and average pore sizes were derived from the adsorption data. Surface elemental composition and chemical states were evaluated by XPS (ESCALAB Xi+, Thermo Fisher Scientific, Waltham, MA, USA). UV-vis DRS spectra were recorded using a UV-2600 spectrometer (Shimadzu Corporation, Kyoto, Japan) with BaSO_4_ as the reflectance standard, and the apparent optical band gaps were estimated from the corresponding Tauc plots. PL spectra were collected using a JEOL JEM-2010F instrument (JEOL Ltd., Tokyo, Japan) at room temperature under an excitation wavelength of 375 nm to evaluate the radiative recombination behavior of photogenerated carriers.

Transient photocurrent response, EIS, and Mott–Schottky measurements were carried out using a CHI660C electrochemical workstation (CH Instruments, Shanghai, China) in a three-electrode configuration under irradiation from a 300 W Xe lamp (CEL-HXF300, Beijing China Education Au-light Co., Ltd., Beijing, China). DRIFTS was employed to monitor the surface intermediates formed during photocatalytic CO_2_ reduction over powder 450 Cu/TiO_2_. The measurements were conducted using a Harrick in situ diffuse reflectance reaction cell (Harrick Scientific Products, Pleasantville, NY, USA) coupled with a Nicolet iS50 FT-IR spectrometer (Thermo Fisher Scientific, Waltham, MA, USA). Typically, 50 mg of catalyst is loaded into the reactor and exposed to a CO_2_/H_2_O mixed atmosphere for 30 min, followed by dark adsorption for 1 h. No TEOA was introduced during the DRIFTS measurements, which were used to probe surface CO_2_ adsorption, activation, and intermediate evolution under simplified in situ conditions. After background subtraction, spectra were recorded with 32 scans at a resolution of 4 cm^−1^. The sample was then irradiated with the same 300 W Xe lamp, and spectral changes were monitored at 10 min intervals.

### 3.4. Photocatalytic CO_2_ Reduction Experiments

The CO_2_ photoreduction activity was evaluated in a sealed batch reactor (CEL-HPR100T+, Beijing China Education Au-light Co., Ltd., Beijing, China) with a 150 mL Teflon liner under irradiation from the same 300 W Xe lamp. The reaction was carried out under continuous stirring, and no additional external heating was applied during irradiation. For each test, 10 mg of the catalyst was dispersed in a mixture of 1 mL TEOA and 4 mL deionized water. Before irradiation, the reactor was purged with high-purity CO_2_ (≥99.999%) for 30 min to remove residual air and ensure a CO_2_-saturated atmosphere. Before light irradiation, the suspension was kept in the dark for 1 h. During irradiation, the reaction mixture was continuously stirred, and gaseous products were sampled every 1 h for 5 h. The gaseous products were quantified by gas chromatography (GC2060-FID, Shanghai Ruimin Instrument Co., Ltd., Shanghai, China) with flame ionization and thermal conductivity detectors. In this study, the product analysis mainly focused on gaseous products, including CH_4_ and CO. To verify the origin of the carbon-containing products, ^13^CO_2_ isotope-labeling tests were performed, and the generated products were detected by gas chromatography–mass spectrometry (GC-MS, 7890A GC/5975C MS, Agilent Technologies, Santa Clara, CA, USA).

## 4. Conclusions

In summary, Cu/TiO_2_ photocatalysts were successfully prepared from Cu-MIL-125 through a temperature-controlled calcination strategy. Structural characterization confirmed that the Cu-MIL-125 precursor was gradually transformed into anatase Cu/TiO_2_, accompanied by the production of porous structures, and formation of surface Cu active sites. The calcination temperature played a crucial role in regulating the crystallinity and interfacial electronic structure of Cu/TiO_2_. Among the prepared samples, 450 Cu/TiO_2_ showed the best overall photocatalytic CO_2_ reduction performance, achieving a CH_4_ formation rate of 15.90 μmol g^−1^ h^−1^ and a CH_4_ selectivity of 93.05%. The improved activity was mainly attributed to the optimized anatase crystallinity, suitable porous structure, effective Cu active sites, enhanced light absorption, and promoted separation and transfer of photogenerated charge carriers. In addition, the unchanged XRD and FT-IR patterns after reaction further confirmed the catalyst maintained good stability during repeated cycling tests. Control experiments demonstrated that light irradiation, photocatalyst, CO_2_, H_2_O and TEOA were essential for the reaction. The ^13^CO_2_ isotope-labeling experiment verified that CH_4_ and CO originated from CO_2_ photoreduction, while in situ DRIFTS revealed the generation and evolution of surface intermediates during CO_2_ activation and hydrogenation. This study offers a practical route for developing MOF-derived Cu/TiO_2_ photocatalysts toward CH_4_-selective CO_2_ photoreduction and highlights calcination-temperature regulation as a key factor in optimizing MOF-derived photocatalysts.

## Figures and Tables

**Table 1:** Physical parameters of materials obtained at different temperatures.

**Samples**	**S_BET_ (m^2^ g^−1^)**	**Pore Volume (cm^3^ g^−1^)**	**Pore Size (nm)**
MIL-125	763.48	0.52	2.74
Cu-MIL-125	135.05	0.23	6.73
300 Cu/TiO_2_	164.35	0.30	7.27
400 Cu/TiO_2_	153.55	0.42	10.85
450 Cu/TiO_2_	104.03	0.44	16.93
500 Cu/TiO_2_	66.64	0.31	18.89
TiO_2_	44.28	0.11	10.07

**Table 2:** Comparison of representative TiO_2_-based photocatalysts for photocatalytic CO_2_-to-CH_4_ reduction.

**Photocatalyst**	**Reaction Conditions**	**CH_4_ Formation Rate**	**Ref.**
TiO_2_/CuInS_2_ hybrid nanofibers	CO_2_ + H_2_O vapor, 350 W Xe lamp	2.50 μmol g^−1^ h^−1^	[40]
TiO_2_/ZnO nanocomposite	CO_2_ + H_2_O, 300 W Xe lamp	2.56 μmol g^−1^ h^−1^	[41]
3% BS/TiO_2_	CO_2_ + H_2_O_2_, continuous-flow photoreactor	4.39 μmol g^−1^ h^−1^	[42]
carbon nanofibers@TiO_2_	CO_2_ + H_2_O, 350 W Xe lamp	13.52 μmol g^−1^ h^−1^	[43]
450 Cu/TiO_2_	CO_2_ + H_2_O + TEOA ^1^, 300 W Xe lamp	15.90 μmol g^−1^ h^−1^	This work

^1^ TEOA = triethanolamine.

## Data Availability

The data supporting the findings of this study are included in the accompanying minimal dataset submitted with this manuscript. Additional data are available from the corresponding author upon reasonable request.
